# Bi-Directional Sexual Dimorphisms of the Song Control Nucleus HVC in a Songbird with Unison Song

**DOI:** 10.1371/journal.pone.0003073

**Published:** 2008-08-27

**Authors:** Manfred Gahr, Reinhold Metzdorf, Dieter Schmidl, Wolfgang Wickler

**Affiliations:** Max Planck Institute for Ornithology, Seewiesen, Germany; Indiana University, United States of America

## Abstract

Sexually dimorphic anatomy of brain areas is thought to be causally linked to sex differences in behaviour and cognitive functions. The sex with the regional size advantage (male or female) differs between brain areas and species. Among adult songbirds, males have larger brain areas such as the HVC (proper name) and RA (robust nucleus of the arcopallium) that control the production of learned songs. Forest weavers (*Ploceus bicolor*) mated pairs sing a unison duet in which male and female mates learn to produce identical songs. We show with histological techniques that the volume and neuron numbers of HVC and RA were ≥1.5 times larger in males than in females despite their identical songs. In contrast, using in-situ hybridizations, females have much higher (30–70%) expression levels of mRNA of a number of synapse-related proteins in HVC and/or RA than their male counterparts. Male-typical and female-typical sexual differentiation appears to act on different aspects of the phenotypes within the same brain areas, leading females and males to produce the same behaviour using different cellular mechanisms.

## Introduction

Sex differences in behaviour, in particular in the realm of reproduction, are common in all vertebrates. In correlation with these behavioural differences, there are many reports of sexual dimorphisms at various organizational levels of the central nervous system of vertebrates including humans [1]–[6]. In particular, the size of brain areas and their neuron numbers have frequently been correlated with functional sex differences [2]–[6]. In songbirds, a chain of forebrain areas including HVC and RA (robust nucleus of the arcopallium) is required for the production of learned vocal pattern [7]–[9]. Activity patterns of the HVC and RA appear to be uniquely associated with song syllable and song element identity, respectively [8]–[9]. These areas seem to differ between males and females in size and neuron numbers in those songbird species in which males and females differ in their vocal behaviour [10]–[12]. There are, however, some reports of the song system and other neurobehavioural models [3], [13]–[15] that do not easily fit this structure-function rule in which more “hardware” is correlated with improved behavioural performance. For example, the African bush shrike (*Lanarius funebris*) has sexually dimorphic sizes and neuron numbers of the song control nuclei HVC and RA as well as sexually dimorphic neuron sizes within HVC, but song complexity is similar in females and males [13]. Such examples suffer, however, from the possibility that there might be subtle sex-differences in the behaviour that are difficult to recognize, which would be in register with their sexually-dimorphic neural phenotype. Indeed, in the bush shrike, although males and females utter similarly-complex songs with similar numbers of syllables, the syllable types are different between mates [13]. However, these small behavioural sex differences are usually thought to relate to small neural sex differences [11], not to large ones as they are found in the bush shrike [13]. Alternatively, this mismatch in the extent of the “neuroanatomy-behaviour” correlation could be due to correlating the wrong entities, since there might be other aspects of the neural phenotype that functionally compensate for the anatomical size difference. For example, the sex with smaller neuron numbers might have more complex network properties, as suggested for the human cortex [3]. To this end we report here on a dueting songbird species, the forest weaver (*Ploceus bicolor*), in which male and female mates sing in unison; they learn to sing an identical song during pair formation [16], [17]. We compared the neuroanatomy of vocal control areas in terms of area size and neuron numbers between male and female mates that were observed to defend their territory with dueting in their natural habitat. Secondly, we compared the expression of a number of genes, in particular sex hormone receptors and synapse related genes, in vocal control areas of these pair mates. Sex differences in gene expression of birds are not reported to be regulated by gene dosage compensation [18] and thus should be higher in males since female birds are the heterogametic sex.

## Results

**Figure 1:**
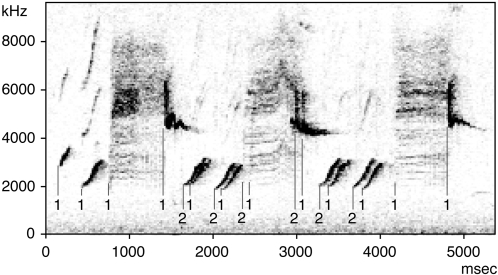
The unison duet of the forest weaver. We show a sonogram of a field recording of a breeding pair of forest weavers from Fannie's Island. To better illustrate the fact that 2 animals are participating with the same syllable repertoire, we selected a duet in which bird 2 sings with a delay of about 90 msec. “1” and “2” indicate the onset of syllables produced by either bird. In many cases the animals duet with an offset of less than 10 msec.

The forest weaver is widespread through Africa south of the Equator in coastal forest [19]. Sexes are indistinguishable in the field, either by eye or ear. Although there are local song dialects that differ in the number of song syllables [20], pair members in all areas studied have an identical song, which is mostly uttered in unison [16], [17]. The species-specific song performance starts with a few flute-like introductory notes, followed by a harsh call-like syllable of rasping quality, which is then followed by a series of clear flute-like melodic syllables (Fig. 1). While the introductory syllables are given only at the beginning of the performance, the song will then go on as a continuous alternation between the harsh syllable and the melodic syllables, sometimes up to more than half a minute without interruption [16], [17]. The repertoire of each pair included in this study was composed of 6 syllables, in agreement with previous investigations of our study population [17]. Due to their unison singing, the singing frequency, repertoire sizes and repertoire composition, i.e. all features of the learned song were similar between males and females as described previously [16], [17] (Fig. 1).

**Figure 2:**
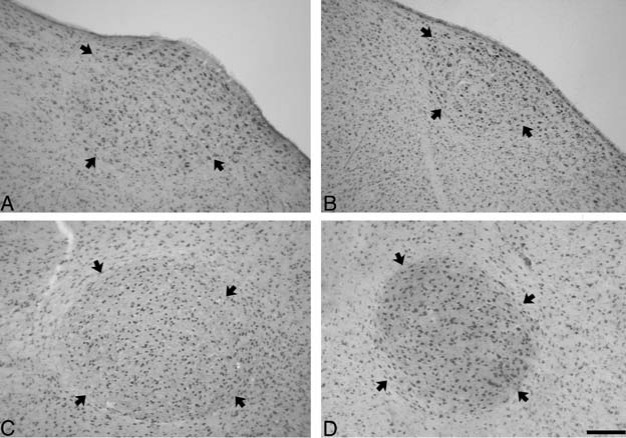
The volume of HVC of all males was larger compared with all females. Photomicrographs of the Nissl-stained HVC (A, B) and RA (C, D) of a male (A, C) and his female (B, D) mate (parasagittal sections). Arrows indicate the borders of HVC and RA. Scale bar is 100 µm.

**Figure 3:**
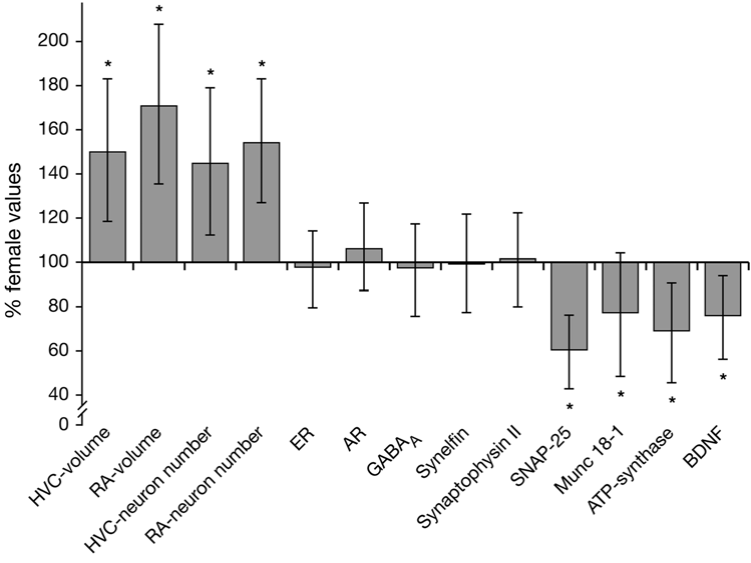
Bi-directional dimorphisms of gross-morphological features (area volume, neuron number) and gene expression of vocal control areas. Data are calculated as male percentages of their female mate (blotted: mean±SD of all pairs (n = 10)). Clearly, the anatomical features (HVC and RA volume and neuron numbers) are much larger in the male, while the expression level of some genes (SNAP-25, Munc-18-1, ATPsynthase, BDNF) is much higher in the female than in the male HVC. * = p-values<0.01 (see text for details).

Morphometric features of vocal control areas (volume, neuron numbers) are frequently thought to correlate sex differences in vocal pattern [10]–[12], although there are exceptions to this hypothesis [13], for review: [21]. In the case of the forest weaver, this dominating notion predicts similar neuroanatomy of male and female mates due to their unison singing. There was no sex difference in the weight of the forebrain, which contains HVC, RA and the entopallium (a visual control region), so we did not correct the anatomical measurements for brain size. The volume of the Nissl-defined HVC and RA was 1.58–1.69 times larger in male compared to female forest weavers (t = −6.577, p<0.0001 for HVC, t = −7.858, p<0.0001 for RA) (Fig. 2 & 3). The volumes (mean (SD)) were 0.53 (0.07) mm^3^ for male HVC, 0.37 (0.08) mm^3^ for female HVC, 0.38 (0.05) mm^3^ for male RA, and 0.23 (0.08) mm^3^ for female RA. Similarly, the size of the HVC defined by AR-mRNA distribution of the males was 1.73 times larger compared to that of the females. Since neuron densities were similar in males and females (p>0.5 for both HVC and RA), males had 1.47 and 1.52 times more HVC- and RA-neurons, respectively (t = −4.461, p = 0.0012 for HVC; t = −5.287, p = 0.0014 for RA). We did not find a sex difference in volume or neuron numbers (p>0.5 for both) of the entopallium (male volumes: 4.87 (0.48) mm^3^; females volumes 4.69 (0.61) mm^3^), a visual forebrain area. Similar data were obtained for the five captive animals included in the study. Both, volumes and neuron numbers were in the same range as those of the wild-caught animals [see Supplementary Results S1]. The data from the captive animals suggest that large sex differences in volumes of song control areas remain in aged animals well beyond their song-learning period since they were at least 3 years old at the time of sacrifice. The ages of the wild-caught birds in this study were not known, but all males and females were observed to duet prior to capturing. The fact that the same neuroanatomical differences were found in both wild-caught and captive animals (who were putatively older) suggests that the smaller vocal control regions of females are not simply a sign of delayed maturation of the female song system.

Messenger RNA (mRNA) of the androgen receptor (AR), estrogen receptor (ER), brain derived nerve growth factor (BDNF), ATPsynthase, GABA_A_ receptor alpha1 subunit, and the synapse related proteins SNAP-25, Munc-18-1, Synaptoporin, and Synelfin was localized in brain sections with radioactive cRNA probes by means of in-situ hybridization. These gene expression studies showed that the number of labeled cells per area was similar, but that the expression level of particular genes was much higher in the HVC and RA of females than in males while the expression of other genes did not differ between mates. In HVC, SNAP-25 (t = 6.804, p<0.0001), Munc-18-1 (t = 3.725, p = 0.0039), BDNF (t = 4.108, p = 0.0021), and ATPsynthase (t = 4.521, p = 0.011) were expressed at higher levels in females than in males (Fig. 3). In RA, only GABA_A_ receptor was expressed more highly in females than in males (t = 4.872, p = 0.0012). The p-values for all other genes of both, the HVC and RA, were not significantly different (p>0.2). We did not find a sex difference in gene-expression in the entopallium for any of the genes. Since SNAP-25 and Munc-18-1 are expressed in basically every HVC neuron and since the expression is homogenous throughout HVC [22]; this study, it is unlikely that the above sex differences are due to particular neuronal subpopulations. Clearly, these sex differences in gene-expression might only be a subset of all such differences and do not exclude the possibility that there are genes with higher expression levels in males than in females. Nevertheless, the subset of genes studied here, which were chosen based on previous knowledge about their expression in vocal areas [22]–[27], runs counter the usual positive correlation between sex, gene expression, and neuron numbers of HVC and RA. In the chicken genome, the ATPsynthase gene is located on a sex chromosome (Z), while all others are located on autosomes; while we don't know the chromosomal locations of the studied genes in the forest weaver, we think it likely that the composition of avian sex chromosomes is conservative. Under normal circumstances, gene expression should be higher in male than in female forest weavers since male birds are the homogametic sex [18]. In contrast with this expectation, the ATPsynthase is more highly expressed in the female forest weaver HVC. It is thus unlikely that the higher patterns of gene expression in female song areas are due to the simple genetics of sex chromosome allocation.

## Discussion

Despite the lack of sex differences in the learned song of forest weaver mates, there is a male-biased (male>female) sexual dimorphism in the anatomy and a female-biased (female>male) sexual dimorphism in gene expression within the vocal control areas HVC and RA that is not seen in a visual control area. This intra-specific dissociation suggests that, (1.) there is no obligate relation between neural morphology and behavioural phenotypes, and (2.) neural sexual dimorphisms indicate sex-specific adaptations to behavioural control rather than sex differences in behaviour. Because the spectral and temporal song features of forest weavers are learned during pair formation [16], [17] these conclusions concern both, mechanisms of song production and of learning in which HVC and RA are involved for review: [28]. Intra-specific dissociations might explain some of the conflicting data in which brain area morphology (volumes, neuron numbers) correlate with the behaviour in certain species but not in others as published for singing and food storing for reviews: [13]–[15], [21], [28]. Such dissociations might also explain why many cognitive functions of men and women are similar, although cortical regions of men appear to contain more neurons while those of females may contain more neuronal processes [3]. Alternatively, none of the above sexual dimorphisms of the song system anatomy and transcription might be related to singing. However, acute injections of BDNF into the RA of adult zebra finches affect the song pattern transiently [29]; under normal circumstances the likely source of BDNF supply to RA is the HVC [25].

The differentiation of song control areas HVC and RA depends both on genetically endowed mechanisms and epigenetic factors such as sex steroid hormones that integrate environmental information [30]–[33]. It is fair to assume that the song control areas of the forest weaver are hormone-sensitive given their songbird-typical expression pattern of androgen and estrogen receptors in HVC and RA (this study). Since singing by female songbirds occurs in several songbird taxa [34], female singing has either been lost or invented independently several times during the songbird radiation. Thus, any evolutionary explanation for the observed bi-directional neural sex difference needs to consider the hormone-dependence of song area development and either a monomorphic or conventional (male more elaborated) dimorphic song phenotype of the ancestors of the forest weaver. In the following scenarios (see below paragraph) we assume for simplicity of the discussion that the genes that are more highly expressed in females are related to synapse density. This assumption is motivated by the large data sets identifying SNAP-25 and Munc-18-1 as core molecules of the synaptic secretory machinery [35] and BDNF as a key player that modulates dendritic branching and synaptic connectivity [36], [37]. The relation of endogenous local expression levels of these genes to synapse numbers and function is widely unknown. Mice heterozygous for a SNAP-25 null mutation have no phenotype [38], while those heterozygous for Munc-18-1 have a smaller pool of readily releasable vesicles [39] and those heterozygous for BDNF null mutation have a reduced stimulus-induced synapse formation [40]. The differences in the gene expression level of such heterozygous and wild-type mice might be in the range of the differences seen in the song control areas of male and female forest weaver.

In the case of a monomorphic (males and females sing) ancestral background, male and female ancestors would have had rather similar song systems, but could have lacked unison dueting. Although females of some other weaver species sing, their song is less complex than that of breeding males or not unison [16], [41]. The sexually size-dimorphic neural phenotype of the forest weaver song system might have evolved due to sex differences in hormonal mechanisms that can be recruited for extending the life-span of vocal learning (unison duets), or for extending the yearly dueting period (facilitating long-term pair maintenance and territoriality). Prolonged periods of elevated sex hormones that facilitate song learning or production might be costly [42], [43], and sex hormone profiles need to differ between mates to avoid negative pleiotropic effects on other hormone-sensitive sexually-dimorphic entities such as the immune system function and egg-laying [21], [42], [43]. In consequence, sex-limited patterns of hormone production would be reflected differently in the differentiation of different phenotypic components (neuron numbers versus synapse density) of the vocal areas, if these phenotypic components differ in their hormone-sensitivity. Indeed, within the HVC, volume and ATPsynthase expression are androgen-sensitive in canaries, while BDNF and synaptoporin expression are estrogen-sensitive in canaries and/or zebra finches [22], [25]–[27]. The present sex differences do not segregate according to this pattern of hormone-sensitivities seen in other species but species differences in hormonal regulation of genes are likely in light of multiple regulatory sites and and/or local abundance of transcription factors.

In case of a dimorphic (females don't sing) ancestral background, it would be reasonable to assume an ancestral sex differences in neuron numbers of song areas, a common feature among current songbirds including the weaver family [12], [13], [41], [44]. Such neuron numbers are brain-intrinsically and hormonally determined [30]–[33]. From other species with sexually dimorphic HVC and RA volumes and neuron numbers, such as the canary, we know that hormone exposure fails to create a monomorphic phenotype [27], [45]. We further know that neuron numbers within HVC are androgen sensitive [27], Hartog and Gahr, unpublished. Extended periods of elevated testosterone level, which might drive neuron recruitment into HVC and HVC-size to an upper limit [26], [27], [45], appear to curtail vocal learning, since such canaries develop abnormal small syllable repertoires [27], [46]. Thus, instead of using an androgen-dependent mechanism to increase neuron numbers, neural capacity in females might be increased by up-regulating the general number of synapses or the numbers of certain types of synapses under the control of estrogens. Interestingly, inter-specific comparison of song system synapse density suggests that the synapse density of female songbirds may be a particularly labile trait [47]. The present study suggests that females and males might solve (in evolutionary terms) the same behavioural problem differently, as proposed for humans [3].

## Methods

### Animals

During the breeding season, we (DS and MG) observed pairs in their territories in Eastern South Africa and recorded the songs of 10 such pairs to assure that both mates were singing and to analyze the syllable repertoires. Males and females of the pairs were subsequently caught and processed for anatomical studies within 30 minutes of recording. The measurements of their body features, gonadal status, breeding and territorial behaviour identified all wild-caught animals as adults. Another 3 males and 2 females, who had been maintained in captivity for up to 12 years at Seewiesen, were also analyzed. The song-learning history of these latter animals was known.

### Song analysis

Vocalizations were recorded with an UHER 4200 stereo tape recorder and an AKG D900C directional microphone. Songs were analyzed with the AVISOFT- SONAGRAPH Pro for WINDOWS.

### Histology

Birds were caught in accordance with permits issued by the local authorities (Chief Professional Officer for Research at the Natal Parks, Game and Fish Preservation Board, P. O. B. 662, Pietermaritzburg 3200). All animals were killed with an overdose of equithesin and perfused transcardially with 0.9% saline followed by 4% phosphate-buffered formaldehyde (PBF). After fixation, we collected morphometric data and removed the brain, gonads and syrinx for post-fixation in PBF for 2 weeks. Brains were then freeze-protected with 10% phosphate-buffered sucrose (1 day) followed by 30% sucrose (1 day) under RNAse free conditions. The left and right halves of each brain were cut with a freezing microtome alternating in five 15 and one 25 µm parasagittal sections per hemisphere under RNAse-free conditions. Sections were collected in RNAse-free phosphate-buffered saline and mounted onto Fisher Superfrost Plus Slides. Each series of 15- and the 25-µm sections was mounted onto different slides so that we obtained six series of adjacent sections. The 15-µm-series were used for in-situ hybridizations, the 25-µm series of the left hemisphere was Nissl-stained with Thionin, and that of the right was NeuN and Nissl-stained. Anatomical measurements (histology, in-situ hybridization) were performed in the forebrain vocal areas HVC and RA and in a forebrain visual area, the entopallium.

### In-situ hybridization

mRNA expressing cells were localized in brain sections with cRNA probes of the zebra finch by means of in-situ hybridization following published protocols [24]. We studied the expression of the estrogen receptor alpha (ER [24]; chicken chromosome (CC) 3), the androgen receptor (AR [24]; CC 4 and micro-chromosome of zebra finches), the neurotrophin BDNF (brain derived nerve growth factor; GenBank DQ086496; CC 5), ATPsynthase alpha subunit (GenBank AF314256; CC Z), GABA_A_-receptor α_1_-subunit (GenBank DQ086494; CC 4), and the synapse related proteins SNAP-25 (synaptosomal-associated protein 25 kDa; GenBank AY531112; CC 3), Munc-18-1 (GenBank DQ086495; CC 17), Synaptoporin (Synaptophysin II; GenBank AY531113; CC 12) and Synelfin (α-synuclein; GenBank DQ086497; CC 4). AR was applied to sections of both hemispheres. ER, BDNF, ATPsynthase and GABA_A_ were applied to sections of the right hemisphere. SNAP-25, Munc-18-1, Synaptophysin, and Synelfin were used with sections of the left hemisphere. For each animal, we used the homologous series of brain sections with a particular gene. Although the above gene fragments were isolated from the zebra finch, they appear to be highly conserved in birds (similarity with the chicken sequences is ≥86% for all probes). The antisense probes all gave positive results in those parts of the forest weaver brain that we expected to be labeled from previous studies of male zebra finches. The sense probes served as controls in preliminary studies, in which we used brains of captive forest weavers. No specific labeling was seen in any of the sense controls.

Antisense or sense RNA probes labeled with ^35^S-CTP (NEN) were generated by transcription of the linearized plasmids containing the appropriate gene sequences (ca 250–800 Bp) using the riboprobe system (Promega) according to the manufacturer's instructions.

### Immunocytochemistry

For the identification of neurons, we stained one series of sections of the right HVC for NeuN, a neuron specific protein, before counterstaining these sections with Thionin. The NeuN protocol was as described [48].

### Morphometric analysis

As there was no sex difference in the weight of the forebrain, which contains HVC, RA and the entopallium, we did not correct the anatomical measurements for brain size. For the estimation of the volumes of brain areas (HVC, RA, entopallium) with an image analysis system (SPOT) we used cytoarchitectural criteria in Nissl-stained brain sections and the distribution of AR-mRNA (Fig. 2). The volume of each brain nucleus was the sum of these measurements (Nissl and AR-mRNA, respectively) multiplied by the section thickness multiplied by the inter-section distance. Since there was no left-right asymmetry of AR-mRNA defined HVC and RA, neuron numbers were only analyzed for the right HVC. Neurons were counted in NeuN-immunostained sections under high power (1000×) with the SPOT-system in ten 2,500 µm^2^-counting frames of each animal and brain area. The total number of neurons was derived from these cell densities and the HVC and RA volume, respectively. Quantification of the in-situ hybridizations was as described [25]. Briefly, we analyzed the percentage of labeled cells in ten 2,500 µm^2^-counting frames per brain area and animal using the 99% Poisson criteria; of the labeled cells, we calculated the average number of silver grains. For statistics we used the paired t-test (n = 10) throughout except the entopallium (n = 9). All data sets were normally distributed. Significant data remained significant after sequential Bonferroni correction for the t-tests (p = 0.01 for HVC and RA volume and neuron numbers, SNAP-25 mRNA of HVC, ATP mRNA of HVC, GABA_A_ of RA; p = 0.05 for BDNF mRNA and Munc-18-1 mRNA of HVC).

## Supporting Information

Results S1 (0.02 MB DOC)

## References

[1] Raisman G, Field P (1971). Sexual dimorphism in the preoptic area of the rat.. Science.

[2] Arnold AP, Gorski R (1984). Gonadal steroid induction of structural sex differences in the CNS.. Ann Rev Neurosci.

[3] de Courten-Myers GM (1999). The human cerebral cortex: gender differences in structure and function.. J Neuropathol Exp Neurol.

[4] Morris JA, Jordan CL, Breedlove SM (2004). Sexual differentiation of the vertebrate nervous system.. Nature Neurosci.

[5] McCarthy MM, Konkle ATM (2005). When is a sex difference not a sex difference?. Front Neuroendocrinol.

[6] Gahr M, Short RV, Balaban E (1994). Brains structure: Causes and consequences of brain sex.. The differences between the sexes.

[7] Nottebohm F, Stokes TM, Leonard CM (1976). Central control of song in the Canary, Serinus canarius.. J Comp Neurol.

[8] Yu AC, Margoliash D (1996). Temporal hierarchical control of singing in birds.. Science.

[9] Hahnloser RHR, Kozhevnikov AA, Fee MS (2002). An ultra-sparse code underlies the generation of neural sequences in a songbird.. Nature.

[10] Nottebohm F, Arnold AP (1976). Sexual dimorphism in vocal control areas of the songbird brain.. Science.

[11] DeVoogd TJ, Brenowitz EA, Arnold AP (1988). Small sex differences in song control dendrities are associated with minimal differences in song capacity.. J Neurobiol.

[12] MacDougall-Shackleton SA, Ball GF (1999). Comparative studies of sex differences in the song-control system of songbirds.. Trends Neurosci.

[13] Gahr M, Sonnenschein E, Wickler W (1998). Sex differences in the size of the neural song control regions in a dueting songbird with similar song repertoire size of males and females.. J Neurosci.

[14] Lucas JR, Brodin A, de Kort SR, Clayton NS (2004). Does hippocampal size correlate with the degree of caching specialization?. Proc Biol Sci.

[15] Bolhuis JJ, Macphail EM (2001). A critique of the neuroecology of learning and memory.. Trends Cogn Sci.

[16] Wickler W, Seibt U (1980). Vocal dueting and the pair bond. II. Unisono dueting of the African forest weaver, Symplectes bicolor.. Z Tierpsychol.

[17] Seibt U, Wickler W, Kleindienst HR, Sonnenschein E (2002). Structure, geography and origin of dialects in the traditive song of the forest weaver Ploceus bicolor sclateri in Natal, S. Africa.. Behaviour.

[18] Itoh Y, Melamed E, Yang X, Kampf K, Wang S (2007). Dosage compensation is less effective in birds than in mammals.. J Biol.

[19] Cyrus DP, Robson NF (1980). Bird atlas of Natal.

[20] Wickler W, Seibt U, Schmidl D (2006). Song differences between populations of seven subspecies of the African forest weaver *Ploceus bicolor* Vieillot (Aves: Passeriformes).. J Ornithol.

[21] Gil D, Gahr M (2002). The honesty of bird song: multiple constraints for multiple traits.. Trends Ecol Evol.

[22] Voigt C, Metzdorf R, Gahr M (2004). Differential expression pattern and steroid hormone sensitivity of SNAP-25 and synaptoporin mRNA in the telencephalic song control nucleus HVC of the zebra finch.. J Comp Neurol.

[23] George JM, Jin H, Woods WS, Clayton DF (1995). Characterization of a novel protein regulated during the critical period for song learning in the zebra finch.. Neuron.

[24] Gahr M, Metzdorf R (1997). Distribution and dynamics in the expression of androgen and estrogen receptors in vocal control systems of songbirds.. Brain Res Bull.

[25] Dittrich F, Feng Y, Metzdorf R, Gahr M (1999). Estrogen-inducible, sex-of brain-derived neurotrophic factor mRNA in a forebrain song control nucleus of the juvenile zebra finch.. Proc Natl Acad Sci U S A.

[26] Louissaint A, Rao S, Leventhal C, Goldman SA (2002). Coordinated interaction of neurogenesis and angiogenesis in the adult songbird brain.. Neuron.

[27] Fusani L, Metzdorf R, Hutchison JB, Gahr M (2003). Aromatase inhibition affects testosterone-induced maculinization of song and the neural song system in female canaries.. J Neurobiol.

[28] Bolhuis J, Gahr M (2006). Neural mechanisms of birdsong memory.. Nat Rev Neurosci.

[29] Kittelberger JM, Mooney R (2005). Acute injections of brain-derived neurotrophic factor in a vocal premotor nucleus reversibly disrupt adult birdsong stability and trigger syllable deletion.. J Neurobiol.

[30] Gahr M, Metzdorf R (1999). The sexually dimorphic expression of androgen receptors in the song nucleus hyperstriatalis ventrale pars caudale of the zebra finch develops independently of gonadal steroids.. J Neurosci.

[31] Nikolakopouloua AM, Parpasa A, Panagisa L, Zikopoulosa B, Dermon CR (2003). Neural, not gonadal origin of brain sex differences in a gynadromorphic finch.. Proc Natl Acad Sci U S A.

[32] Gahr M (2004). Hormone-dependent neural plasticity in the juvenile and adult song system: what makes a successful male?. Ann N Y Acad Sci.

[33] Wade J, Arnold AP (2004). Sexual differentiation of the zebra finch song system.. Ann N Y Acad Sci.

[34] Langemore NE (1998). Functions of duet and solo songs of female birds.. Trends Ecol Evol.

[35] Lang T, Jahn R (2008). Core proteins of the secretory machinery.. Handb Exp Pharmacol.

[36] Horch HW (2004). Local effects of BDNF on dendritic growth.. Rev Neurosci.

[37] Bramham CR, Messaoudi E (2005). BDNF function in adult synaptic plasticity: the synaptic consolidation hypothesis.. Prog. Neurobiol.

[38] Washbourne P, Thompson PM, Carta M, Costa ET, Mathews JR (2001). Genetic ablation of the t-SNARE SNAP-25 distinguishes mechanisms of neuroexocytosis.. Nat Neurosci.

[39] Toonen RFG, Wierda K, Sons MS, de Wit H, Cornelisse LN (2006). Munc18-1 expression levels control synapse recovery by regulating readily releasable pool size.. Proc Natl Acad Sci USA.

[40] Gernoud C, Knott GW, Sakata K, Lu B, Welker E (2004). Altered synapse formation in the adult somatosensory cortex of brain-derived neurotrophic factor heterozygote mice.. J Neurosci.

[41] Voigt C (2005). Inter- and intrasexual dimorphism in the song and song control system of duetting White-browed sparrow weavers..

[42] Searcy WA (1988). Do female red-winged blackbirds limit their own breeding densities?. Ecology.

[43] Rutkowska J, Cichon M, Puerta M, Gil D (2005). Negative effects of elevated testosterone on female fecundity in zebra finches.. Hormon Behav.

[44] Voigt C, Leitner S, Gahr M (2007). Socially induced brain differentiation in a cooperatively breeding songbird.. Proc R Soc B.

[45] Nottebohm F (1980). Testosterone triggers growth of brain vocal control nuclei in adult female canaries.. Brain Res.

[46] Hartley RS, Chinn MS, Ullrich NF (1997). Left syringeal dominance in testosterone-treated female canaries.. Neurobiol Learn Mem.

[47] McNealen P (2005). An interspecific comparison using immunofluorescence reveals that synapse density in the avian song system is related to sex but not to male song repertoire size.. Brain Res.

[48] Stamatakis A, Barbas H, Dermon CR (2004). Late granule cell genesis in quail cerebellum.. J Comp Neurol.

