# Supplementary material for: Bi-Directional Sexual Dimorphisms of the Song Control Nucleus HVC in a Songbird with Unison Song
Source: PLoS One. 2008 Aug 27;3(8):e3073. doi: 10.1371/journal.pone.0003073 (PMC2518117; doi:10.1371/journal.pone.0003073)
Supplement: Results S1 — (0.02 MB DOC) [file pone.0003073.s001.doc]

**Results S1 Supplementary Information: Results**

**Neuroanatomy of captive animals:**

Neuroanatomical data similar to those of the wild caught birds were obtained for the five captive animals included in the study. Both, volumes and neuron numbers were in the same range as those of the wild-caught animals; as example we include the volumes of the Nissl-defined areas: male HVCs: 0.461, 0.538, 0.597 mm^3^, female HVCs: 0.335, 0.245 mm^3^, male RA: 0.369, 0.325, 0.283 mm^3^, female RA: 0.192, 0.137 mm^3^.
